# Plasma p‐tau231 in the presenilin 1 E280A autosomal dominant Alzheimer’s disease kindred: a cross‐sectional and longitudinal cohort study

**DOI:** 10.1002/alz.086532

**Published:** 2025-01-09

**Authors:** Vincent Malotaux, Nicholas J. Ashton, Annika Öhrfelt, Yinghua Chen, Yi Su, Gloria Garcia, Claudia F Guzman, Andres Villegas‐Lanau, Margarita M. Giraldo‐Chica, David Fernando Aguillon, Victoria Tirado, Ana Y. Baena, Claudia Muñoz, Natalia Acosta‐Baena, Jeremy J. Pruzin, Valentina Ghisays, Silvia Ríos‐Romenets, Clara Vila‐Castelar, Jairo E. Martinez, Averi Giudicessi, Pierre N. Tariot, Henrik Zetterberg, Francisco Lopera, Kaj Blennow, Eric M. Reiman, Yakeel T. Quiroz

**Affiliations:** ^1^ Massachusetts General Hospital, Harvard Medical School, Boston, MA USA; ^2^ Department of Psychiatry and Neurochemistry, Institute of Neuroscience and Physiology, University of Gothenburg, Mölndal, Gothenburg Sweden; ^3^ Department of Psychiatry and Neurochemistry, Institute of Neuroscience and Physiology, University of Gothenburg, Mölndal Sweden; ^4^ Banner Alzheimer's Institute, Phoenix, AZ USA; ^5^ Grupo de Neurociencias de Antioquia, Universidad de Antioquia, Medellín Colombia; ^6^ University of Arizona, Phoenix, AZ USA; ^7^ Clinical Neurochemistry Laboratory, Sahlgrenska University Hospital, Mölndal Sweden; ^8^ Grupo de Neurociencias de Antioquia, Facultad de Medicina, Universidad de Antioquia, Medellín Colombia; ^9^ Translational Genomics Research Institute, Phoenix, AZ USA; ^10^ Arizona State University, Phoenix, AZ USA

## Abstract

**Background:**

Plasma tau phosphorylated at threonine 231 (p‐tau231) is a promising novel biomarker of emerging Alzheimer's disease (AD) pathology. We aimed to characterize cross‐sectional and longitudinal plasma p‐tau231 measurements and estimated ages of biomarker onset in an exceptionally large number of presenilin (PSEN1) E280A (Glu280Ala) mutation carriers and age‐matched non‐carriers from the Colombian autosomal dominant Alzheimer’s disease kindred.

**Method:**

We included a cohort of 722 PSEN1 E280A mutation carriers (mean age 36.36± 12.43 years; 399 females) and 640 non‐carriers (mean age=37.29±13.29 years; 350 females) with baseline assessments from the Alzheimer’s Prevention Initiative Registry at the University of Antioquia (Medellín, Colombia); of these participants, longitudinal measures were available for 164 mutation carriers and 132 non‐carriers (with a mean follow‐up of 4.8±3.1 and 5.6±3.2 years respectively). We used an ultrasensitive Single molecule array (Simoa) for the quantification of plasma p‐tau231 developed at the University of Gothenburg. Using log‐transformed data, we examined the relationship between plasma p‐tau231 levels and age to establish the earliest age at which p‐tau231 concentrations begin to diverge between mutation carriers and non‐carriers. Rates of change in p‐tau231 levels were also compared between the two groups. Data were modeled using Linear Mixed Effects Models, a restricted cubic spline, & Hamiltonian Markov chain Monte Carlo analyses.

**Result:**

Carriers had higher levels of plasma p‐tau231, compared to non‐carriers (9.05±7.44 vs. 5.21±3.37, p<0.001). Plasma p‐tau231 measurements were significantly correlated with age in both groups (r=0.63 in carriers vs. r=0.19 in non‐carriers, p<0.001) and began to differentiate carriers from non‐carriers at age 23 (21 years before the estimated median age at mild cognitive impairment [MCI] onset for this kindred). In population with longitudinal data, PSEN1 E280A mutation carriers had a higher rate of change in p‐tau231 levels compared to non‐carriers (0.03±0.02 vs. 0.01±0.01, p<0.001) and began to differ from non‐carriers at age 19, 25 years before the carriers’ estimated median age of MCI onset.

**Conclusion:**

Our findings support the promise of plasma p‐tau231 as a biomarker for the detection and tracking of AD pathology, the identification of potential candidates for clinical trials and the evaluation of disease‐modifying therapies.

